# Supplementary material for: Tissue factor-dependent colitogenic CD4+ T cell thrombogenicity is regulated by activated protein C signalling
Source: Nat Commun. 2025 Feb 16;16:1677. doi: 10.1038/s41467-025-57001-7 (PMC11830781; doi:10.1038/s41467-025-57001-7)
Supplement: Supplementary file 1 — Supplementary Information [file 41467_2025_57001_MOESM1_ESM.pdf]

SUPPLEMENTARY DATA

Tissue factor-dependent colitogenic CD4^{+} T cell thrombogenicity is regulated by activated protein C signaling.

Dr. Gemma Leon….Prof. Roger J.S. Preston*.

***Corresponding Author:**
Roger Preston PhD
Irish Centre for Vascular Biology,
School of Pharmacy and Biomolecular Sciences,
RCSI University of Medicine and Health Sciences,
Dublin, Ireland.
Email: rogerpreston@rcsi.ie
ORCID: https://orcid.org/0000-0003-0108-4077

SUPPLEMENTARY FIGURES

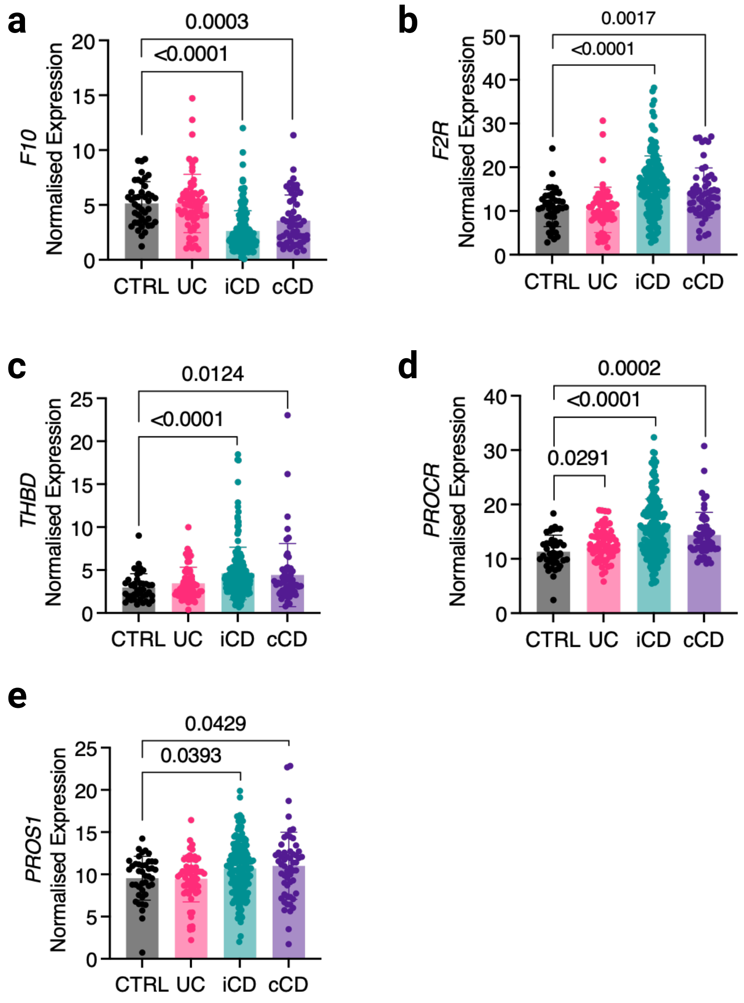

Supplementary Figure 1: Coagulation genes are dysregulated in IBD. Expression of **(a)** *F10*, **(b)** *F2R*, **(c)** *THBD*, **(d)** *PROCR* and **(e)** *PROS1* from RNA-seq data of colonic biopsies from Crohns Disease (CD; illeal (iCD, n=162), colonic (cCD, n=56), Ulcerative Colitis (UC, n=62), and query IBD healthy control cohort (CTRL, n=42) patients in the RISK study (GEO ID GSE57945). Mann Whitney U Test **(a-e)** was used to determine and expressed as mean mean ± s.d.

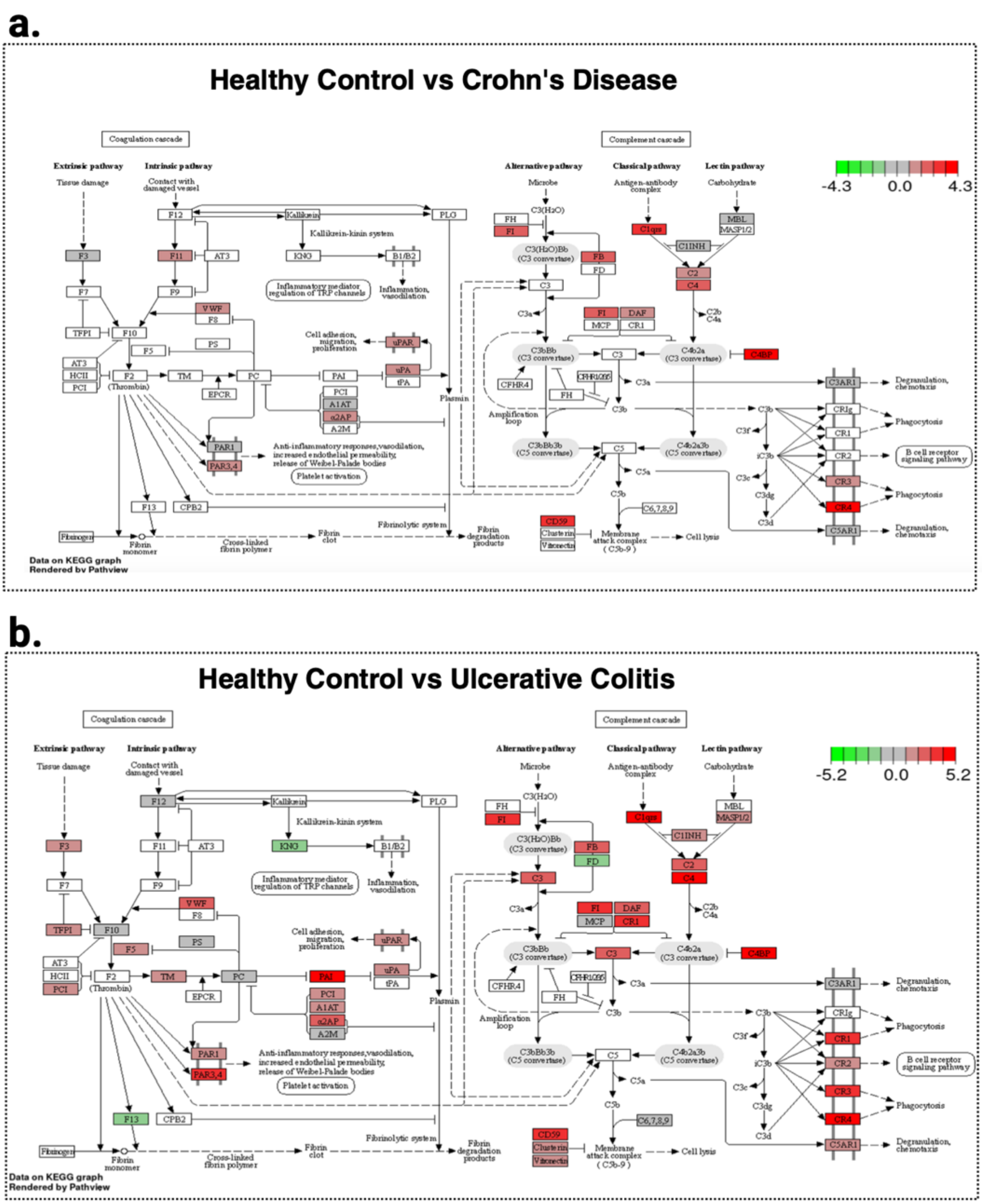

Supplementary Figure 2: Coagulation pathways are dysregulated in IBD patients colons. KEGG pathway analysis was used to depict dysregulation in the coagulation pathway in RNA-seq samples of colonic biopsies from paediatric CD patients compared to **(a)** healthy controls and **(b)** paediatric UC patients CD, n=11; UC, n = 7; Ctrl, n = 12.

a

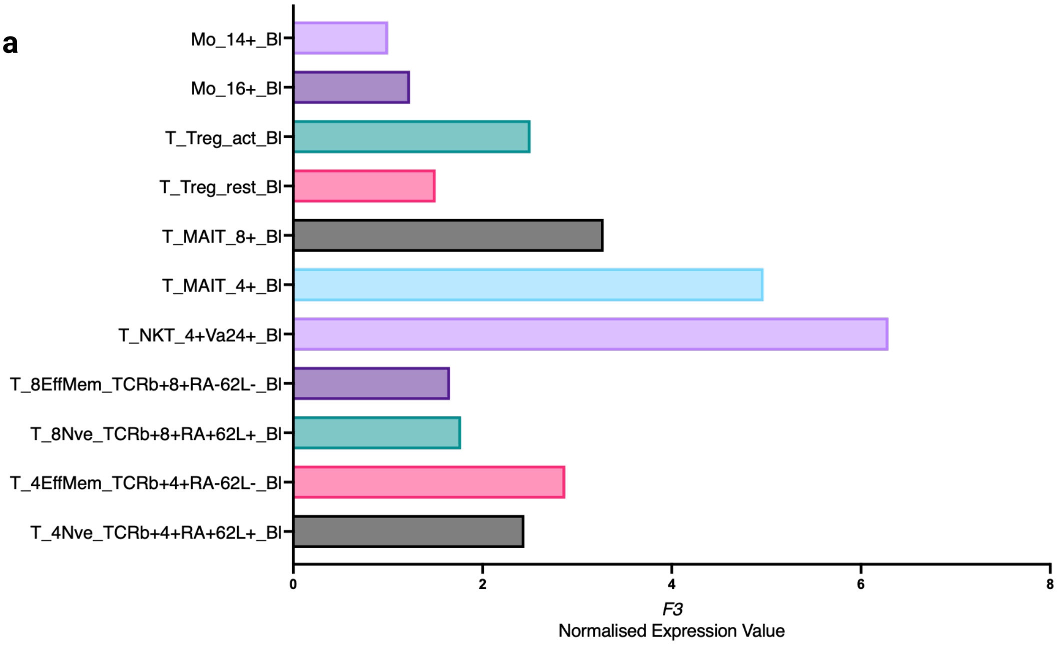

b

Population Name	Organ	Cell Type	Description
T.4Nve.CD3+4+RA+62L+.Bl	Blood	abT	Sorted as DR- TCRb+ CD4+ CD8- CD127+ CD25- CD54RAhi CD62Lhi PBMCs
T.4EffMem.CD3+4+RA-62L-.Bl	Blood	abT	Sorted as DR- TCRb+ CD4+ CD8- CD127+ CD54RA- CD62L- PBMCs
T.8Nve.CD3+8+RA+62L+.Bl	Blood	abT	Sorted as DR- TCRb+ CD4- CD8+ CD127+ CD25- CD54RAhi CD62Lhi PBMCs
T.8EffMem.CD3+8+RA-62L-.Bl	Blood	abT	Sorted as DR- TCRb+ CD4- CD8+ CD127+ CD54RA- CD62L- PBMCs
T.NKT.Va24+.Bl	Blood	abT	Sorted as CD19- CD3+ XXX PBMCs
T.MAIT.4+.Bl	Blood	abT	Sorted as CD19- CD3+ CD4+ CD8- Va72.2+ CD161dull PBMCs
T.MAIT.8+.Bl	Blood	abT	Sorted as CD19- CD3+ CD4- CD8+ Va72.2+ CD161dull PBMCs
T.Treg.rest.Bl	Blood	abT	Sorted as CD19- CD4- CD25+ CD127- CD45RA+ PBMCs
T.Treg.act.Bl	Blood	abT	Sorted as CD19- CD4- CD25+ CD127- CD45RA- PBMCs
Mo.16+.Bl	Blood	Mo	Sorted as HLA.DR+ CD16hi CD14lo PBMCs
Mo.14+.Bl	Blood	Mo	Sorted as HLA.DR+ CD16lo CD14hi PBMCs

Supplementary Figure 3: *F3* expression by human immune cells. (a) TF expression in human immune cells contributing to IBD pathogenesis was analysed using the Human Cell Atlas of RNA-seq datasets uploaded to the Immunological Genome Project (http://immunecellatlas.net). **(b)** A descriptive table of immune cell populations and their markers was included in the analyses.

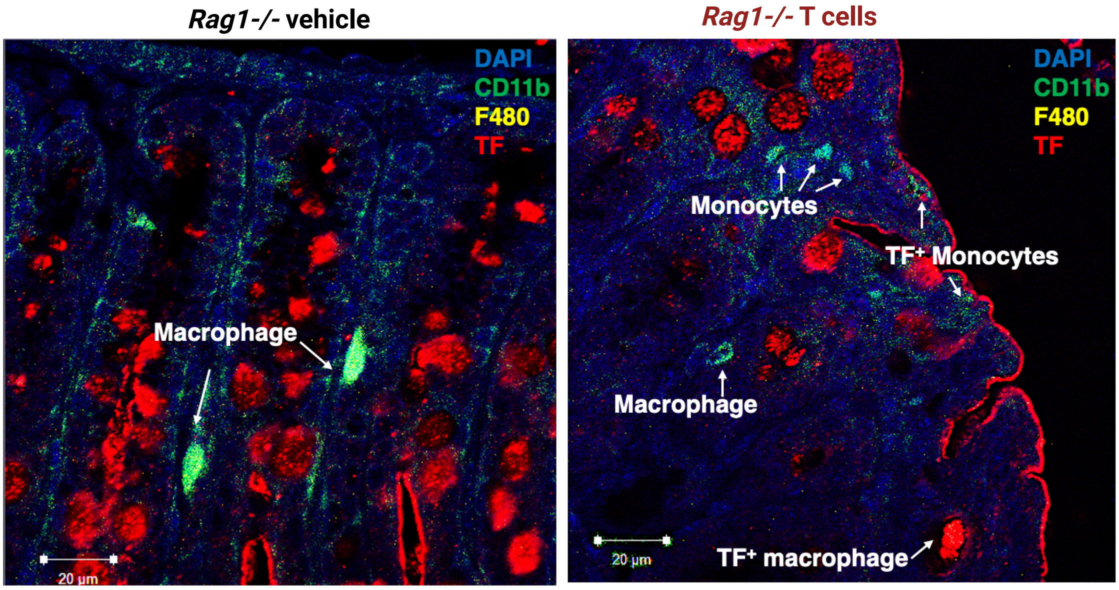

Supplementary Figure 4: The presence of infiltrating monocytes/macrophages was assessed by performing TF, CD11b and F480 co-staining of formalin-fixed mouse colons following T cell transfer-induced colitis (reproduced n = 4 *Rag1*$^{-/-}$ T cells and n = 3 *Rag1*$^{-/-}$ vehicle).

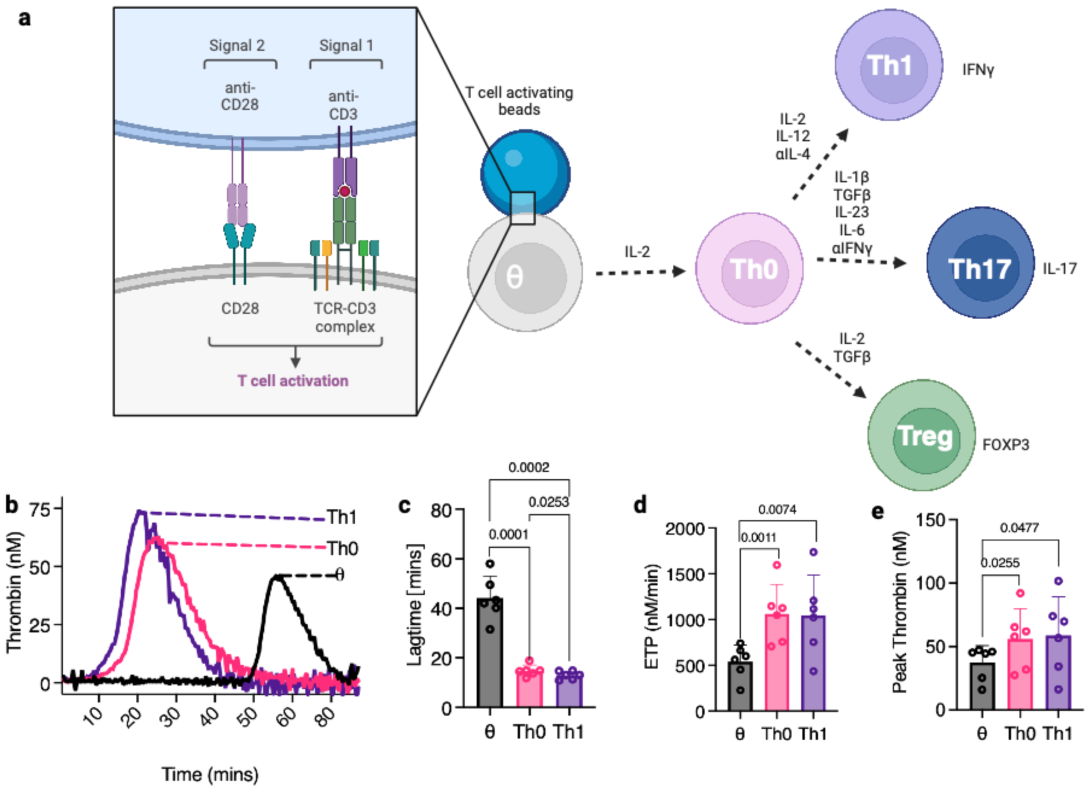

Supplementary Figure 5: CD4+ T cell-mediated thrombin generation in FXII-deficient plasma: (a) Schematic diagram representing the process of T cell activation and differentiation requirements. CD4+ T cells require 2 signals to activate fully. They first require stimulation of the T cell receptor (TCR/CD3)('signal 1') by Major histocompatibility complex (MHC) Class 2 molecules presented by antigen-presenting cells or by anti-CD3 molecules presented on the surface of synthetic beads. Stimulation of the T cell co-stimulatory receptor CD28 is then required ('signal 2'), either by CD86 presented by antigen-presenting cells or by anti-CD28 molecules presented on the surface of synthetic beads. In our experiments, we used anti-CD3/anti-CD28 coated beads to activate T cells fully. Once activated, IL-2 is required to enhance viability and proliferation, and specific cytokines are applied to skew the activated Th0 cells towards distinct inflammatory lineages (Th1: IL-2, αIL-4, IL-12, Treg: IL-2, TGFβ, Th17: TGFβ, IL-1β, IL-23, & IL-6, αIFNγ). **(b-e)** Calibrated automated thrombinography was used to measure extrinsic pathway-mediated T cell procoagulant activity using FXII-deficient plasma. **(c)** Lagtime, **(d)** endogenous thrombin potential (ETP) and **(e)** peak thrombin levels were measured and compared between θ (unactivated cells), Th0 and Th1 cells**.** Students paired t-test (two-tailed) **(c-e)** was used to determine statistical significance and expressed as mean ±s.d for 6 biological replicates. Created in BioRender. Preston, R. (2025) https://BioRender.com/y45l979.

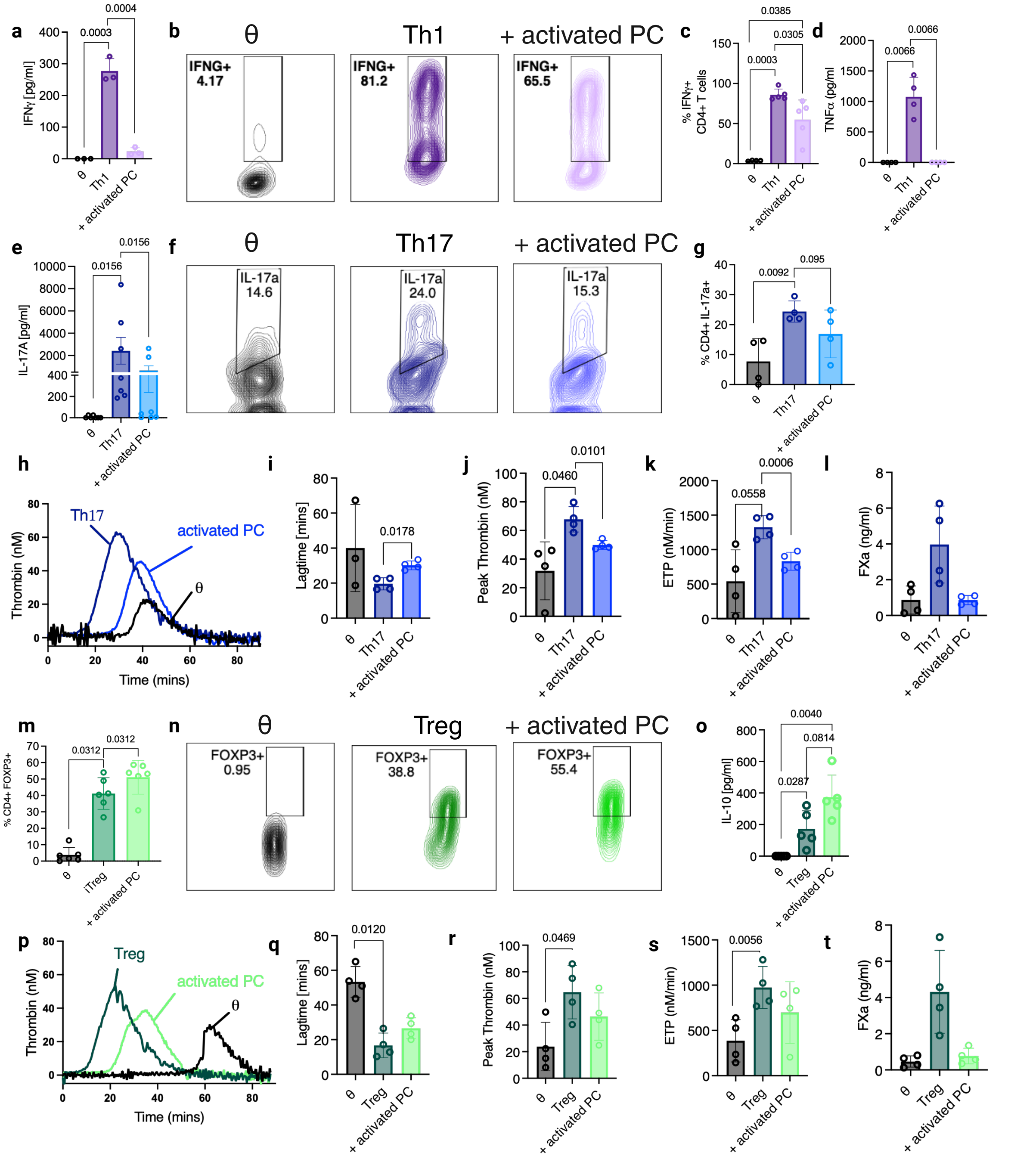

Supplementary Figure 6: Activated PC regulates both CD4+ T proinflammatory activity and Th17 and iTreg thrombogenicity. (a-t) CD4+ T cells were isolated from donor human blood and plated at a density of 0.8x10^6/ml with IL-2 for unactivated conditions (θ). Plated cells were activated with the addition of αCD3/αCD28 activation beads and stimulated with IL-2 for Th0 conditions, differentiation cytokines were added to skew cells to a **(a-d)** Th1 lineage (αIL-4 + IL-12, a **(e-l)** Th17 lineage (TGFβ, IL-1β, IL-23, & IL-6, αIFNγ) or **(m - t),** iTreg lineage (IL-2, TGFβ), and cells were treated +/- 20nM activated PC for 5 days. In Th1 cells pre-treated with activated PC (**a**) IFNγ levels and **(d)** TNFα levels were measured by ELISA, and **(b & c)** the percentage of IFNγ+ cells was measured by flow cytometry. Using Th17 cells pre-treated with activated PC **(e)** IL-17a levels were measured by ELISA, and **(f & g)** the percentage of IL-17a+ cells was measured by flow cytometry. The percentage of FOXP3+ cells was measured

by flow cytometry in Treg cells that had been previously treated with activated PC **(m & n),** and IL-10 levels were measured by ELISA **(o)**. Calibrated automated thrombinography was used to measure extrinsic pathway-mediated T cell procoagulant activity using FXII-deficient plasma in Th17 and iTreg cells treated with activated PC **(h-k, p-s)**. **(i & q)** Lagtime, **(j & r)** peak thrombin levels and **(k & s)** ETP were measured, as was their capacity to facilitate FXa generation **(l & t)**. Students t-test (two-tailed) **(a),** Students paired t-test (two-tailed) **(c, d, g, i-k, o, & q-s)** or Wilcoxon test (two-tailed) **(e & m)** was used to determine statistical significance and expressed as mean ± S.D. **(a, c, d, g, i-k, m, o, & q-s)** or mean ± S.E.M. **(e)** for **(a)** 3, **(c & o)** 5, **(d, g, i-k & q-t**) 4, **(e)** 7, and **(m)** 6 biological replicates.

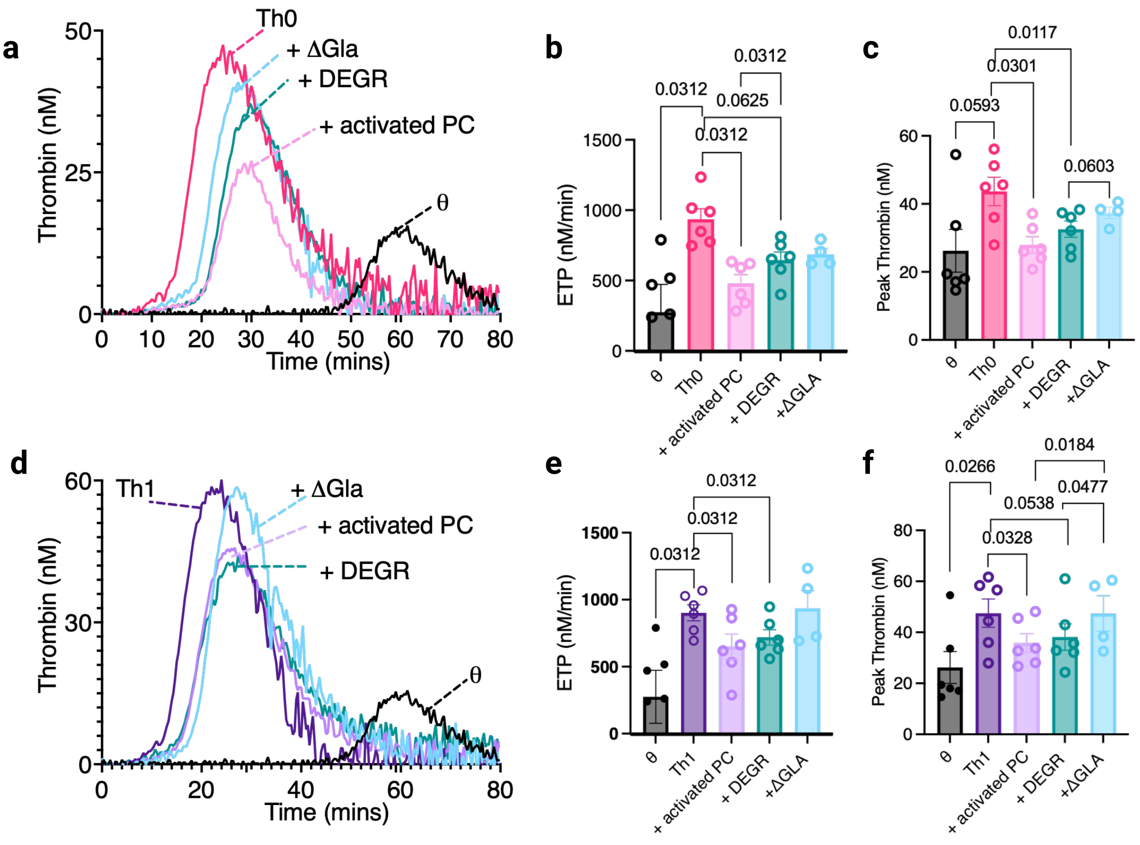

Supplementary Figure 7: Role of EPCR binding and activated PC amidolytic activity in inhibiting TF-mediated T cell procoagulant activity. To assess the role of EPCR and PAR signalling in activated PC-mediated inhibition of T cell thrombogenicity, Gla-domainless activated PC (**Δ**GLA) or active site-blocked activated PC (Dansyl-EGR-chloromethlyketone (DEGR) were employed. θ (unactivated cells), Th0 and Th1 cells were grown in culture in media supplemented with activated PC, activated $PC^{\Delta GLA}$ or activated PC^{DEGR} for 5 days. Following incubation, cells were washed with EDTA containing PBS and calibrated automated thrombinography was used to measure TF-mediated T cell procoagulant activity in FXII-deficient plasma **(a-f)**. **(b & e)** ETP and **(c & f)** peak thrombin levels were measured and compared. Students paired t-test (two-tailed) **(c & f)** or Wilcoxon test (two-tailed) **(b & e)** was used to determine statistical significance and expressed as mean ± S.E.M. for 4-6 biological donors(n = 6 θ, Th0/Th1, + activated PC, activated PC^{DEGR}; n=4 activated $PC^{\Delta GLA}$).

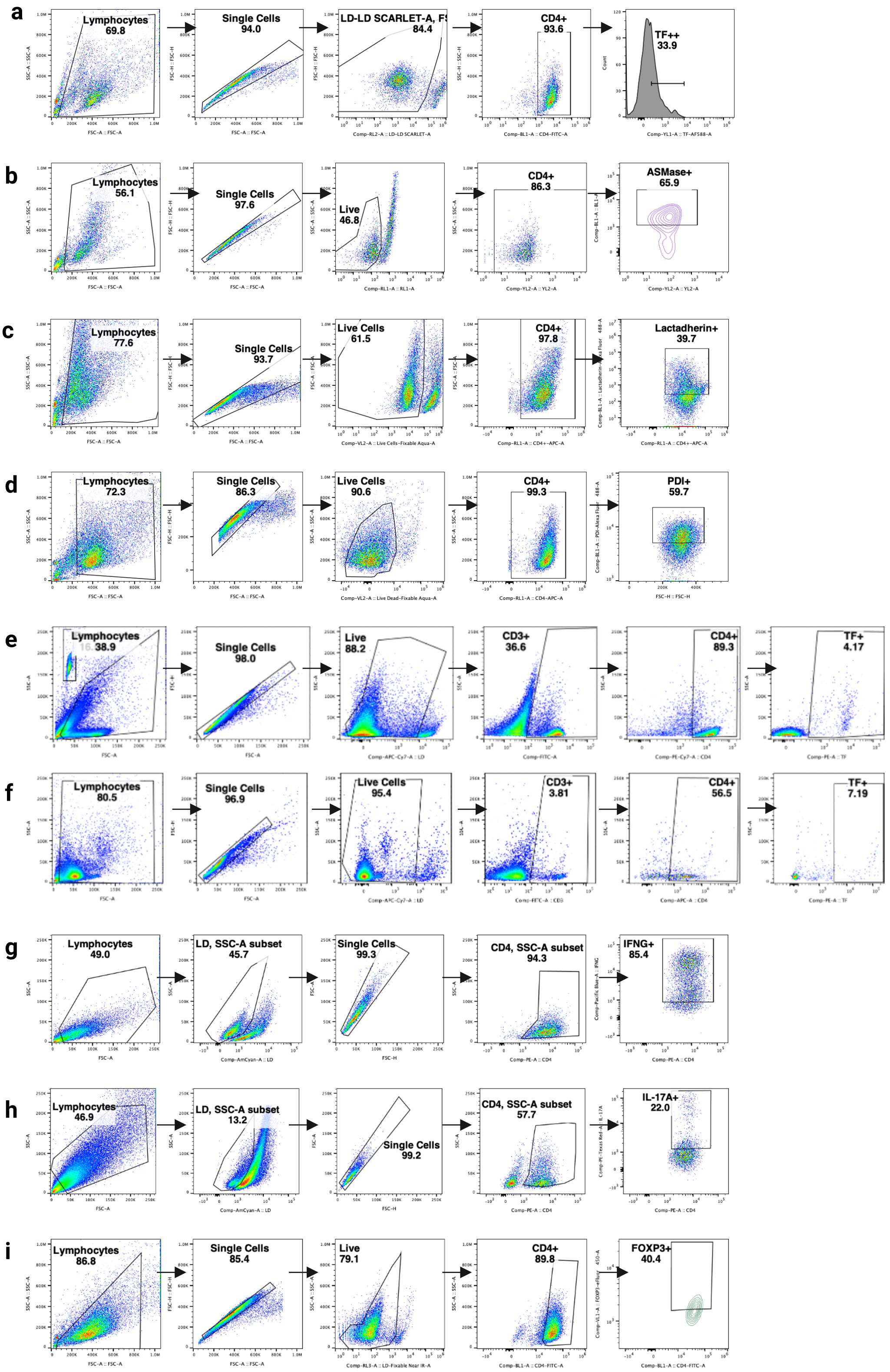

Supplementary Figure 8: Flow cytometry gating strategies. Gating strategies for flow cytometry experiments **(a)** Figure 3k-m **(b)** Figure 4b&c **(c)** Figure 4e&f **(d)** Figure 4h&I, Figure 6r-u **(e)** Figure 1e-h **(f)** Figure 5a-e **(g)** Supplementary Figure 6 b&c **(h)** Supplementary Figure 6 f&g **(i)** Supplementary Figure 6 n&o**.**

SUPPLEMENTARY TABLES

Supplementary Table 1: Antibodies and staining reagents.

Antibody/Dye	Congugate	Species Reactivity	Clone	Company	Catalogue Number
**CD4**	PE	Human	RPA-T4	eBioscience, Thermofisher	12-0049-42
**CD4**	FITC	Human	RPA-T4	eBioscience, Thermofisher	11-0049-42
**CD3**	FITC	Human	UCHT1	eBioscience, Thermofisher	1-0038-80
**CD3**	FITC	Mouse	145-2C11	eBioscience, Thermofisher	11-0031-82
**IFNg**	PE	Human	4S.B3	eBioscience, Thermofisher	12-7319-42
**IFNg**	eFluor 450	Human	4S.B3	eBioscience, Thermofisher	48-7319-42
**F480**	APC	Mouse	BM8	eBioscience, Thermofisher	17-4801-82
**CD11b**	FITC	Mouse	M1/70	eBioscience, Thermofisher	11-0112-82
**PDI (Monoclonal Mouse IgG2a)**		Human, Mouse, Rat, Hamster, Pig	RL90	Invitrogen, Thermofisher	# MA3-019
**CD16/CD32**		Human, Mouse, Rat, Dog, Hamster	93	eBioscience, Thermofisher	14-0161-81
**TF (Polyclonal Goat IgG)**		Human		R&D	AF2339-SP
**TF (Polyclonal Goat IgG)**		Mouse		R&D	AF3178-SP
**Donkey anti-Goat IgG (H+L) Cross-Adsorbed Secondary Antibody**	Alexa Fluor 555	Goat		Invitrogen, Thermofisher	A-21432
**ASM Polyclonal Antibody (Polyclonal Rabbit IgG)**		Human, Mouse, Rat		Invitrogen, Thermofisher	PA5-77047
**Donkey anti-Mouse IgG (H+L) Highly Cross-Adsorbed Secondary Antibody**	Alexa Fluor 488	Mouse		Invitrogen, Thermofisher	A-21202
**Invitrogen Goat anti-Rabbit IgG (H+L) Highly Cross-Adsorbed Secondary Antibody**	Alexa Fluor Plus 488	Rabbit		Invitrogen, Thermofisher	A32731TR

**Invitrogen Goat anti-Rabbit IgG (H+L) Highly Cross-Adsorbed Secondary Antibody**	Alexa Fluor Plus 555	Rabbit		Invitrogen, Thermofisher	A-21429
**LIVE/DEAD Fixable Near IR (780) Viability Kit**	Near-IR (780)			Invitrogen, Thermofisher	L34994
**LIVE/DEAD Fixable Aqua Dead Cell Stain Kit**	Aqua (405)			Invitrogen, Thermofisher	L34957
**LIVE/DEAD Fixable Scarlet (723) Viability Kit**	Scarlet (723)			Invitrogen, Thermofisher	L34986
**Invitrogen Mouse IgG2a Isotype Control (PPV-04)**		Mouse	PPV-04	Invitrogen, Thermofisher	MA1-10419
**Invitrogen Mouse IgG1 kappa Isotype Control**	FITC	Mouse	P3.6.2.8.1	eBioscience, Thermofisher	11-4714-81
**Invitrogen Armenian Hamster IgG Isotype Control**	FITC	Armenian Hamster	eBio299Arm	eBioscience, Thermofisher	11-4888-81
**Invitrogen Mouse IgG1 kappa Isotype Control**	PE	Mouse	P3.6.2.8.1	eBioscience, Thermofisher	12-4714-82
**Invitrogen Mouse IgG1 kappa Isotype Control**	eFluor 450	Mouse	P3.6.2.8.1	eBioscience, Thermofisher	48-4714-80
**APC Rat IgG1, κ Isotype Ctrl Antibody**	APC	Rat	RTK2071	Biolegend	400411

Supplementary Table 2: Patient Information Table for RNA-seq and T Cell Studies.

**Patient Information**		**Numbers**
**Disease Groups**	UC	11
	CD	19
	CTRL	14
**Sex**	Female	9
	Male	13
**UC Phenotype**	E1	0
	E2	3
	E3	0
	E4	9
**CD Phenotype**	L1	1
	L2	12
	L3	5
	L4a	6
	L4b	1
	PA disease only	0
	Oral Disease	1

References:

1. Smillie CS, Biton M, Ordovas-Montanes J, et al. Intra- and Inter-cellular Rewiring of the Human Colon during Ulcerative Colitis. *Cell*. 2019;178(3):714-730.e22.
