# Supplementary material for: Tissue factor-dependent colitogenic CD4+ T cell thrombogenicity is regulated by activated protein C signalling
Source: Nat Commun. 2025 Feb 16;16:1677. doi: 10.1038/s41467-025-57001-7 (PMC11830781; doi:10.1038/s41467-025-57001-7)
Supplement: Supplementary file 2 — Reporting Summary [file 41467_2025_57001_MOESM2_ESM.pdf]

Corresponding author(s): Roger Preston

Last updated by author(s): 17/1/25

Reporting Summary

Nature Portfolio wishes to improve the reproducibility of the work that we publish. This form provides structure for consistency and transparency in reporting. For further information on Nature Portfolio policies, see our Editorial Policies and the Editorial Policy Checklist.

Statistics

For all statistical analyses, confirm that the following items are present in the figure legend, table legend, main text, or Methods section.

n/a	Confirmed	
☐	☒	The exact sample size (*n*) for each experimental group/condition, given as a discrete number and unit of measurement
☐	☒	A statement on whether measurements were taken from distinct samples or whether the same sample was measured repeatedly
☐	☒	The statistical test(s) used AND whether they are one- or two-sided<br>*Only common tests should be described solely by name; describe more complex techniques in the Methods section.*
☐	☒	A description of all covariates tested
☐	☒	A description of any assumptions or corrections, such as tests of normality and adjustment for multiple comparisons
☐	☒	A full description of the statistical parameters including central tendency (e.g. means) or other basic estimates (e.g. regression coefficient) AND variation (e.g. standard deviation) or associated estimates of uncertainty (e.g. confidence intervals)
☐	☒	For null hypothesis testing, the test statistic (e.g. *F*, *t*, *r*) with confidence intervals, effect sizes, degrees of freedom and *P* value noted<br>*Give P values as exact values whenever suitable.*
☒	☐	For Bayesian analysis, information on the choice of priors and Markov chain Monte Carlo settings
☐	☒	For hierarchical and complex designs, identification of the appropriate level for tests and full reporting of outcomes
☒	☐	Estimates of effect sizes (e.g. Cohen's *d*, Pearson's *r*), indicating how they were calculated

Our web collection on statistics for biologists contains articles on many of the points above.

Software and code

Policy information about availability of computer code

Data collection	We utilised multi-omic data from publicly available datasets of IBD patient intestinal biopsies, including RNA-seq data from the Risk Stratification and Identification of Immunogenetic and Microbial Markers of Rapid Disease Progression in Children with Crohn's Disease (RISK) study, in which we compared the expression of genes of interest between paediatric CD and UC patients and healthy controls. We accessed NCBI Gene Expression Omnibus datasets using GEO ID GSE57945 (Risk Cohort).<br><br>We also utilised in-house RNA-seq data generated from paediatric IBD patients and control participants' biopsies recruited in the DOCHAS study at the Children's Health Ireland (CHI) gastroenterology unit (GEO ID GSE266325).
Data analysis	For the RISK cohort (GEO ID GSE57945) The log2 fold-change and p-value significance data were downloaded and analysed using GraphPad Prism 9.5 software. Data are shown as means ± SEM.<br><br>For the paediatric IBD patient cohort (GEO ID GSE266325) a 150bp Paired End strategy was employed for sequencing. Raw data was filtered using fastp software to remove low quality reads prior to downstream analysis. Paired end clean reads were aligned to the reference genome using Hisat2 v2.0.5. For the quantification of gene expression levels featureCounts v1.5.0-p3 was

used and Fragments Per Kilobase of transcript per Millions base pairs sequenced (FPKM) was calculated. Relative expression levels for selected genes of interest between patient groupings were analysed based upon mean FPKM values for each group. Relative gene expression per individual gene is shown as z scores in a heatmap. KEGG pathway analysis was used to depict dysregulation in the coagulation pathway.

For manuscripts utilizing custom algorithms or software that are central to the research but not yet described in published literature, software must be made available to editors and reviewers. We strongly encourage code deposition in a community repository (e.g. GitHub). See the Nature Portfolio guidelines for submitting code & software for further information.

Data

Policy information about availability of data

All manuscripts must include a data availability statement. This statement should provide the following information, where applicable:

- Accession codes, unique identifiers, or web links for publicly available datasets
- A description of any restrictions on data availability
- For clinical datasets or third party data, please ensure that the statement adheres to our policy

All data included in the Supplementary Information are available from the authors, as are any unique reagents used in this article. The raw numbers for charts and graphs are available in the Source Data file whenever possible. The RNA-seq data analysed in this study from paediatric IBD patients is available from the NCBI Gene Expression Omnibus database under accession code GSE266325 (https://www.ncbi.nlm.nih.gov/geo/query/acc.cgi?acc=GSE266325). RNA-seq data analysed in this study from IBD patients in the RISK cohort is available from the NCBI Gene Expression Omnibus database under accession code GSE57945 (https://www.ncbi.nlm.nih.gov/geo/query/acc.cgi?acc=GSE57945).

Research involving human participants, their data, or biological material

Policy information about studies with human participants or human data. See also policy information about sex, gender (identity/presentation), and sexual orientation and race, ethnicity and racism.

Reporting on sex and gender	While the sex of the DOCHAS participants has been recorded as part of the clinical data, we did not stratify data to analyse sex based differences. As these are a paediatric cohort of patients Information on gender has not been collected.
Reporting on race, ethnicity, or other socially relevant groupings	We did not report on race, ethnicity or socially relevant groupings. Our clinical data was derived from an Irish cohort of paediatric patients.
Population characteristics	Our clinical data was derived from an Irish cohort of male and female treatment naive paediatric patients.
Recruitment	All human samples were obtained with consent/assent from paediatric IBD patients and control participants recruited in the Determinants and Outcomes of CHildren and Adolescents with IBD Study (DOCHAS) at the gastroenterology unit at Children's Health Ireland (CHI), Crumlin (Dublin, Ireland). All participants underwent diagnostic evaluation according to international paediatric standards (Porto criteria) and rigorously phenotyped using the paediatric-specific Paris classification of IBD. Rectal and colonic biopsies were obtained from patients enrolled in the study. Patients initially enrolled with suspected IBD, but subsequently not diagnosed with disease, comprise the control population.
Ethics oversight	All experiments using DOCHAS IBD tissues were performed under approval from the institutional Research Ethics Committee (Childrens Health Ireland, Crumlin, Dublin)(GEN/193/11), and included 45 participants (RNA-seq experiments: CD n=9; UC, n = 5; Ctrl, n = 9 CD,T cell experiments: CD n=10; UC, n = 7; Ctrl, n = 5).

Note that full information on the approval of the study protocol must also be provided in the manuscript.

Field-specific reporting

Please select the one below that is the best fit for your research. If you are not sure, read the appropriate sections before making your selection.

☒ Life sciences ☐ Behavioural & social sciences ☐ Ecological, evolutionary & environmental sciences

For a reference copy of the document with all sections, see nature.com/documents/nr-reporting-summary-flat.pdf

Life sciences study design

All studies must disclose on these points even when the disclosure is negative.

Sample size	For DOCHAS IBD cohort experiments:<br>A sample of total 22 control and IBD participants was used for immunofluoresence experiments, T cell TF expression and thrombogenicity studies. Post-hoc analysis revealed this sample size to have a power of 100%, with the probability of a type 1 error set at a=0.05.<br>A sample size of 11 CD patients, 7 UC patients and 12 control participants was used for RNA-seq studies. Post-hoc analysis revealed this sample size to have a power of 100%, with the probability of a type 1 error set at a=0.05<br><br>For mice studies a sample size 3 control PBS vehicle control mice and 4 T effector cell recipient mice was uses. Post-hoc analysis revealed this

	sample size to have a power of 88.7%, with the probability of a type 1 error set at a=0.05.
Data exclusions	No data were excluded.
Replication	For all in vitro experiments 4-10 biological replicates were used.
Randomization	Randomisation was not performed as clinical diagnoses needed to be segregated for analyses
Blinding	Blinding was performed for analyses of mouse and human imaging, and T cell TF expression and thrombogenicity studies conducted with IBD patients. The experiments were conducted by the researcher who knew the grouping of the participants. This researcher psuedoanonmised the results by assigning the samples a study number. Scoring was conducted by a separate researcher who did not know the groupings.

Reporting for specific materials, systems and methods

We require information from authors about some types of materials, experimental systems and methods used in many studies. Here, indicate whether each material, system or method listed is relevant to your study. If you are not sure if a list item applies to your research, read the appropriate section before selecting a response.

Materials & experimental systems

n/a	Involved in the study	
☐	☒	Antibodies
☒	☐	Eukaryotic cell lines
☒	☐	Palaeontology and archaeology
☐	☒	Animals and other organisms
☒	☐	Clinical data
☒	☐	Dual use research of concern
☒	☐	Plants

Methods

n/a	Involved in the study	
☒	☐	ChIP-seq
☐	☒	Flow cytometry
☒	☐	MRI-based neuroimaging

Antibodies

Antibodies used

*CD4 PE Human, RPA-T4 eBioscience, Thermofisher, 12-0049-42
*CD4 FITC Human, RPA-T4, eBioscience, Thermofisher, 11-0049-42
*CD3 FITC Human, UCHT1, eBioscience, Thermofisher, 1-0038-80
CD3 FITC Mouse, 145-2C11, eBioscience, Thermofisher, 11-0031-82
IFNg PE Human, 4S.B3, eBioscience,Thermofisher, 12-7319-42
IFNg eFluor 450, Human 4S.B3 eBioscience, Thermofisher, 48-7319-42
PDI (MonoclonalMouse IgG2a)Human, Mouse, Rat,Hamster, Pig, RL90 Invitrogen, Thermofisher, # MA3-019
CD16/CD32 Human, Mouse, Rat, Dog, Hamster, 93 eBioscience, Thermofisher, 14-0161-81
F480 APC Mouse BM8 eBioscience, Thermofisher 17-4801-82
CD11b FITC Mouse M1/70 eBioscience, Thermofisher 11-0112-82
TF (Polyclonal Goat IgG) Human, R&D, AF2339-SP
TF (Polyclonal Goat IgG) Mouse, R&D, AF3178-SP
Donkey anti-Goat IgG (H+L) Cross-Adsorbed Secondary Antibody Alexa Fluor 555 Goat, Invitrogen, Thermofisher, A-21432
ASM Polyclonal Antibody (Polyclonal Rabbit IgG) Human, Mouse, Rat, Invitrogen, Thermofisher, PA5-77047
Donkey anti- Mouse IgG (H+L) Highly Cross-Adsorbed Secondary Antibody Alexa Fluor 488, Mouse, Invitrogen, Thermofisher, A-21202
Invitrogen Goat anti-Rabbit IgG (H+L) HighlyCross-Adsorbed Secondary Antibody Alexa Fluor Plus 488 Rabbit Invitrogen, Thermofisher, A32731TR
Invitrogen Goat anti-Rabbit IgG(H+L) Highly Cross-Adsorbed Secondary Antibody Alexa Fluor Plus 555 Rabbit Invitrogen, Thermofisher A-21429
Invitrogen Mouse IgG2a Isotype Control (PPV-04) Mouse PPV-04 Invitrogen, Thermofisher MA1-10419
Invitrogen
Mouse IgG1 kappa Isotype Control, FITC Mouse P3.6.2.8.1 eBioscience, Thermofisher, 11-4714-81
Invitrogen Armenian Hamster IgG Isotype Control FITC Armenian Hamster eBio299Arm eBioscience, Thermofisher 11-4888-81
Invitrogen Mouse IgG1, kappa Isotype Control PE Mouse P3.6.2.8.1 eBioscience, Thermofisher, 12-4714-82
Invitrogen
Mouse IgG1 kappa Isotype Control eFluor 450, Mouse P3.6.2.8.1 eBioscience, Thermofisher, 48-4714-80
APC Rat IgG1κsotype Ctrl Antibody, APC, Rat, RTK2071, Biolegend, 400411

Validation

*These antibodies was verified by Relative expression to ensure that the antibody binds to the antigen stated.
CD3 145-2C11 antibody has been tested by immunohistochemistry on frozen mouse spleen.
IFN gamma Antibody (12-7319-42) verified by Cell treatment to ensure that the antibody binds to the antigen stated
IFN gamma Monoclonal Antibody (4S.B3) verified by Cell treatment to ensure that the antibody binds to the antigen stated.
PDI Monoclonal Antibody (RL90) was verified by Knockdown to ensure that the antibody binds to the antigen stated.
CD16/CD32 Antibody (14-0161-82) was verified by flow cytometry.

Animals and other research organisms

Policy information about studies involving animals; ARRIVE guidelines recommended for reporting animal research, and Sex and Gender in Research

Laboratory animals	C57BL/6 male mice between 6-8 weeks of age were used as T cell donors in T cell transfer colitis experiments. As male mice were used as donors, only male Rag1-/- mice between 8-10 weeks of age were used as T cell recipients and PBS vehicle control recipients in T cell transfer experiments. This is because while XX antigen is accepted by both male and female recipients, the Y chromosome and male antigens are rejected by female recipients.
Wild animals	This study did not involve wild animals.
Reporting on sex	C57BL/6 male mice between 6-8 weeks of age were used as T cell donors in T cell transfer colitis experiments. As male mice were used as donors, only male Rag1-/- mice between 8-10 weeks of age were used as T cell recipients and PBS vehicle control recipients in T cell transfer experiments. This is because while XX antigen is accepted by both male and female recipients, the Y chromosome and male antigens are rejected by female recipients.
Field-collected samples	This study did not involve field collected samples.
Ethics oversight	The Health Products Regulatory Authority approved all animal experiments under Project License AE19136/P125.

Note that full information on the approval of the study protocol must also be provided in the manuscript.

Plants

Seed stocks	N/A
Novel plant genotypes	N/A
Authentication	N/A

Flow Cytometry

Plots

Confirm that:

- [ ] The axis labels state the marker and fluorochrome used (e.g. CD4-FITC).
- [ ] The axis scales are clearly visible. Include numbers along axes only for bottom left plot of group (a 'group' is an analysis of identical markers).
- [ ] All plots are contour plots with outliers or pseudocolor plots.
- [x] A numerical value for number of cells or percentage (with statistics) is provided.

Methodology

Sample preparation

Anonymised healthy donor buffy coats were obtained from the Irish Blood Transfusion Service, St. James' Hospital, Dublin. Paediatric IBD and non-IBD control blood samples were obtained from the DOCHAS study, National Centre for Paediatric Gastroenterology, CHI-Crumlin, Dublin, Ireland. PBMCs were isolated using Lymphoprep density gradient centrifugation (STEMCELL Technologies), and magnetic selection was then used to purify CD4+ T cells (CD4 T cell isolation Kit, human, Miltenyi Biotec). Isolated CD4+ T cells were plated at a density of 0.8x106/ml in AIM media supplemented with CTS Immune Cell SR (Gibco, ThermoFisher), activated with anti-CD3/anti-CD28 activation beads (Gibco Dynabeads Human T-Activator CD3/CD28 for T-Cell Expansion and Activation, Thermofisher), stimulated with IL-2 (R&D), and/or T cell differentiating cytokines and antibodies (Th1: anti-IL-4, IL-12 (Miltenyi Biotec), Treg: TGFβ (Immunotools), Th17: TGFβ, IL-1β (R&D), IL-23 (R&D), IL-6 (R&D), anti-IFNγ (Miltenyi Biotec)) +/- APC (Cambridge ProteinWorks) and incubated at 37°C for 5-7 days.

Paediatric IBD and non-IBD biopsies were obtained from the DOCHAS study, National Centre for Paediatric Gastroenterology, CHI-Crumlin, Dublin Ireland. Lamina propria cells were isolated from these biopsies using a modified version of the Smillie et al Star Method1. 4 biopsies per patient were collected in AIM media (ThermoFisher) supplemented with CTS Immune Cell SR (Gibco, ThermoFisher). Biopsies were first rinsed in 30ml of ice-cold PBS (Sigma Aldrich, Merck) and then transferred to 10ml epithelial cell solution (HBSS Ca/Mg-Free 10mM EDTA) (Sigma Aldrich, Merck), 100 U/ml penicillin (ThermoFisher), 100 mg/ml streptomycin (ThermoFisher), 10mM HEPES (Sigma Aldrich, Merck), and 2% FCS (ThermoFisher) freshly supplemented with 200µl of 0.5M EDTA. The epithelial layer was separated from the underlying lamina propria by shaking horizontally at

37°C for 15 min at 200rpm. The tube was then placed on ice for 10 minutes and shaken vigorously 15 times. The tissues were carefully removed and placed into 10ml of ice-cold PBS to rinse, and transferred to 5ml of enzymatic digestion mix (RPMI1640 (ThermoFisher), 100 U/ml penicillin, 100mg/ml streptomycin, 10mM HEPES, 2% FCS, 50 mg/ml gentamicin (ThermoFisher), 100mg/ml of Liberase TM (Roche, Merck) and 100 mg/ml of DNase I (Sigma Aldrich, Merck). The tubes were shaken horizontally at 37°C for 30 min at 200 rpm. After 30 minutes, 1ml of FCS and 80ml of 0.5M EDTA were added to quench the digestion mix and the tubes were placed on ice for five minutes. The dissociated lamina propria cell solution was then filtered through a 40µm cell strainer into a new 50 ml conical tube and rinsed through with PBS. The tube was then spun down at 400g for 10 minutes, and the pelleted live cells were counted using Trypan blue exclusion (ThermoFisher). Cells were resuspended at 1x106/ml in AIM-V media supplemented with CTS Immune Cell SR (Gibco, ThermoFisher), and incubated at 37°C for 24-48hrs prior to analyses by flow cytometry.

Following cell culture staining commenced. All fluorescently-labelled antibodies were purchased from Thermofisher. Cell viability was measured using the LIVE/DEAD Fixable Dead Cell Stain Kit (Aqua, Scarlet & Near IR) (Invitrogen, Thermofisher). Before staining, cells were incubated with an anti-CD16/CD32 monoclonal antibody to block Fc receptors (Invitrogen). Cells were washed using PBS and incubated with LIVE/DEAD dye for 30 minutes at 4oC. Cells were washed using PBS supplemented with 2% FBS and then incubated with fluorescently labelled antibodies for 1 hour at 4oC. For unconjugated antibodies (αASM (Invitrogen), αPDI (Invitrogen)& αTF (R&D)), cells were incubated for 1 hour at room temperature with the primary antibody, washed and incubated with a specific secondary antibody for 1 hour at 4oC (Donkey anti-Goat IgG (H+L) Cross-Adsorbed Secondary Antibody Alexa Fluor 555 and Invitrogen Goat anti-Rabbit IgG (H+L) Highly Cross-Adsorbed Secondary Antibody Alexa Fluor 555, Thermofisher). Fluorescence Minus One (FMO) and/or isotype controls were used to assess positive staining. Intracellular protein expression was evaluated by first restimulating the cells with phorbol-12-myristate 13-acetate (PMA; 10 ng/ml) (Sigma Aldrich), Ionomycin (1 ug/ml) (Sigma Aldrich) and Brefeldin A (5 ug/ml) (eBioscience) for 4–6 h at 37 °C. A FOXP3 staining buffer set (eBiosciences) was used in accordance with the manufacturer's instructions to fix and permeabilise cells after surface staining to facilitate the detection of intracellular cytokines.Intracellular fluorescently labelled antibodies were incubated for 1 hour at 4oC.

Instrument

Flow cytometry was performed on a LSR/Fortessa (BD) or Attune Nxt (Thermofisher). Cell sorting was performed on a Melody (BD).

Software

FlowJo 10 software.

Cell population abundance

For cell sorting experiments of donor C57B6 T effector cells, CD4+ CD45Rbhigh CD25low cells were sorted from a population of magnetically enriched CD4+ T cells using a BD melody. Population purity was between 98-100%.
For flow cytometry experiments of human magnetically enriched CD4+ T cells, cell purity was determined by CD4+ expression in each experiment. Live cell populations exhibited 80-100% CD4+ purity.

Gating strategy

For cell sorting experiments of donor C57B6 T effector cells the gating strategy was. FSC-A vs SSC-A -> FSC-A vs FSC-H (Single Cells) -> SSC-A vs Live Dead stain (Live Cells) -> SSC-A vs CD4-A (CD4+ population) -> CD45Rb vs CD25 (CD45Rbhigh CD25low T effector cells).
For human T cell flow cytometry experiments the typical gating strategy was FSC-A vs SSC-A -> FSC-A vs FSC-H (Single Cells) -> SSC-A vs Live Dead stain (Live Cells) -> SSC-A vs CD4-A (CD4+ population) -> ASMase-A/TF-A/Lactadherin-A/IL-17a-A,IFNg-A,FOXP3-A vs CD4-A (final plotted population).

Fluorescence minus one (FMO), isotype controls and isoclonic controls were used to define gates via negative space gating.

A figure containing the gating strategy for all experiments is provided in Supplementary Figure 8.

☒ Tick this box to confirm that a figure exemplifying the gating strategy is provided in the Supplementary Information.
